# Design and testing of a compact triple differential pumping chamber for gas attenuators

**DOI:** 10.1107/S1600577526009033

**Published:** 2026-10-27

**Authors:** Hao Zhang, Lei Li, Hanwen Lin, Yukai Liu, Wei Wei, Xuan Liu, Feng Zhao, Qinming Li, Weiqing Zhang

**Affiliations:** a Institute of Advanced Light Source Facilities, Shenzhen, Guangdong518107, People’s Republic of China; b Dalian Institute of Chemical Physics Chinese Academy of Sciences Dalian Liaoning116023 People’s Republic of China; RIKEN SPring-8 Center, Japan

**Keywords:** gas attenuators, free-electron laser, differential pumping system, triple differential pumping chamber, 3D printing

## Abstract

The gas attenuator (GAT) is a crucial device to attenuate the intensity of the free-electron laser for Shenzhen Superconducting Soft X-ray Free-electron Laser (S^3^FEL). In most current GATs, high-pumping-speed mechanical pumps have been employed as the first stage of the differential pumping system (DPS) to achieve the working pressure transition. Thus, a compact triple differential pumping chamber (TDPC) based on 3D printing was designed in our preliminary research of GATs to realize a compact, rapid and cost-effective pressure transition. The simulation results reveal that it yields good differential capability with three low-speed scroll pumps (8 L s^−1^) and efficient differential performance from 200 Pa to 22.3 Pa, which facilitates operation of the next differential pump (turbomolecular pump). Moreover, the experimental results show a vacuum leakage rate of 1.69 ×10^−12^ Pa m^3^ s^−1^ and a relative error of less than 25% compared with the simulated pressure. Furthermore, the gas injection position of the active gas cell and the heating temperature (600°C) near the differential pipe have a negligible effect on the differential performance of the TDPC. This study demonstrates the novel concept of a differential pumping chamber, reflects its application prospects in DPS, and provides a useful reference for the DPS design of GATs.

## Abstract 2

This paper presents a triple differential pumping chamber based on 3D printing and machining to realize a compact, rapid, and cost-effective pressure transition for the differential pumping system of gas attenuators. Finite element analysis methods and experimental tests were employed to verify its differential performance. The results reflect its application prospects in differential pumping systems, providing a useful reference for the differential pumping system design of gas attenuators.

## 1. Introduction

Free-electron lasers (FELs) are characterized by high brightness, ultrashort pulses, high coherence and continuously adjustable wavelengths. They have demonstrated significant advantages in fundamental research across fields such as physics, chemistry and biology, thereby, earning the reputation as a ‘high-speed camera’ for elucidating the microstructure and dynamics of matter (Huang *et al.*, 2021 ▸). Several free-electron laser devices have been constructed or are under development worldwide, including EXFEL, LCLS-II, SACLA, DCLS, and SHINE (Corno *et al.*, 2020 ▸; Zhang *et al.*, 2020 ▸; Tanaka *et al.*, 2018 ▸; Wang *et al.*, 2025 ▸; Meng *et al.*, 2024 ▸). The S^3^FEL is a world-leading fourth-generation advanced light source currently in the planning and construction phases, and will be the first soft X-ray free-electron laser device capable of operating at a megahertz repetition frequency (Xing *et al.*, 2025 ▸).

In the beamline design of S^3^FEL, it is crucial to attenuate the intensity of the FEL generated by the undulators to safeguard the downstream optical equipment. Solid attenuators, such as those made from metal or graphite, feature high attenuation accuracy and stability without introducing optical distortions or other interferences but suffer from high mechanical stress and fatigue damage (Xie *et al.*, 2024 ▸). Moreover, gas attenuators (GATs) can provide flexible and continuously adjustable attenuation capabilities while maintaining beam characteristics during operation (Hahn & Tiedtke, 2007 ▸). Its operating principle involves the attenuation of photon intensity through the interaction between gas molecules and photons, enabling varying attenuation magnitudes to be achieved by adjusting the gas pressure. Common working gases comprise nitrogen (N_2_), argon (Ar) and krypton (Kr). For GATs, the attenuation effect under ideal conditions can be calculated by the Beer–Lambert law (Ackermann *et al.*, 2007 ▸), 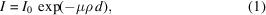
where *I*_0_ (W cm^−2^) and *I* (W cm^−2^) are the input and output photon intensity, respectively. *d* (cm) is the length that can be approximated as the length of the active gas cell, μ (cm^2^ g^−1^) is the mass absorption cross section, depending on the gas type. ρ (g cm^−3^) is the density, which is related to pressure.

The working pressure of a GAT can be calculated from the ideal gas law, 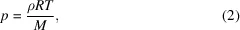
where *p* is the working pressure of the GAT (Pa), ρ (g cm^−3^) the density, *R* the ideal gas constant (J mol^−1^ K^−1^), *T* the temperature (K) and *M* the molar mass of the gas (kg mo1^−1^).

The GAT system is primarily composed of a windowless active gas cell (AGC), a gas injection system and a differential pumping system (DPS) at both its upstream and downstream. The AGC is filled with a specific attenuation gas by the gas injection system, and its pressure is adjusted to a prescribed value (ranging from tens to thousands of pascals) according to equations (1) and (2), depending on the AGC length, the required attenuation and the gas type. Meanwhile, the DPS provides the required pressure drop to the average beamline pressure (< 1.3 × 10^−6^ Pa) and lowers the gas flow from the AGC to the upstream and downstream high-vacuum equipment in the beamline.

FLASH, the world’s first soft X-ray high repetition rate FEL, was the first FEL to develop a GAT, using a turbo­molecular pump as the first-stage differential pump in the DPS with a maximum AGC pressure of 10 Pa (Tiedtke *et al.*, 2009 ▸). Regarding higher pressure of the AGC, high-pumping-speed mechanical pumps (roots pumps or screw pumps) have been typically utilized as the first-stage differential pumps in the DPS of the GATs to achieve the transition from working pressure to the next turbomolecular pumps, as observed in facilities such as EuXFEL, LCLS-II and SHINE (Dommach *et al.*, 2021 ▸; Feng *et al.*, 2016 ▸; Wang *et al.*, 2024 ▸). Nonetheless, these applications demonstrate several disadvantages, such as high cost, complex maintenance requirements, significant vibration and noise, and substantial space occupancy.

The GAT, as an essential device of the beamline in S^3^FEL, is currently under development for preliminary experimental studies. In this paper, a compact triple differential pumping chamber (TDPC) was proposed based on 3D printing technology to achieve rapid, cost-effective pressure transition and a compact design in the GAT. Additionally, finite element analysis (FEA) methods were employed to verify the differential performance (from 200 Pa to 22.3 Pa) of the TDPC with three low-speed scroll pumps (8 L s^−1^). The experiment yielded a vacuum leakage rate of 1.69 × 10^12^ Pa m^3^ s^−1^ for the TDPC and reflected a pressure drop from 200 Pa to 29 Pa, which verifies the favourable consistency (a relative error within 25%) and facilitates the operation of the next differential pump (turbomolecular pump). Simultaneously, the gas injection position of the AGC and the heating temperature near the differential pipe have a negligible effect on the differential performance of the TDPC. This study presents a novel concept of the differential pumping chamber (DPC), demonstrates its application prospects in DPS, and guides the DPS design of GATs.

## 2. Differential pumping theory

### 2.1. Principle of the DPS

**Figure 1:**
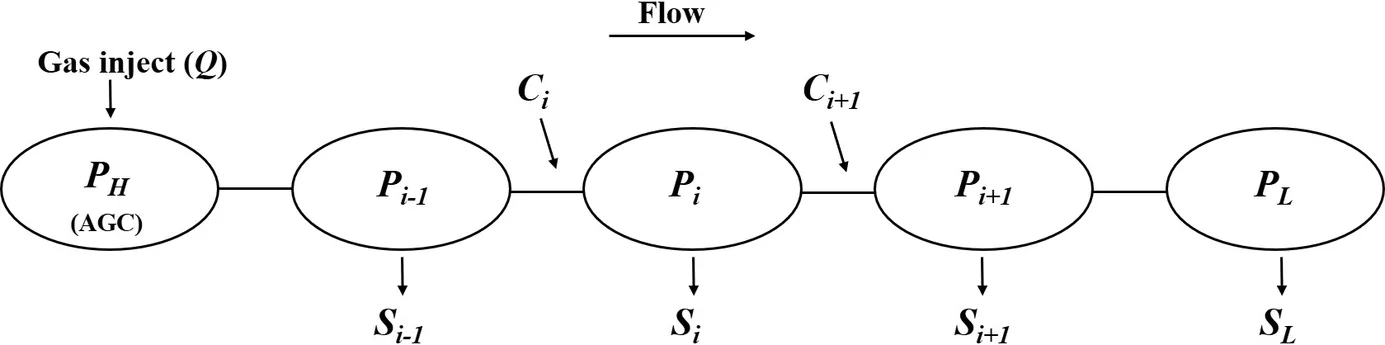
Principle of the DPS in the GAT.

The DPS is used to realize the pressure transition from high pressure to low pressure, following the core principle of flow conservation. Fig. 1 ▸ illustrates the principle of the DPS in the GAT. The flow continuity balance can be expressed as (Wang *et al.*, 2023 ▸): 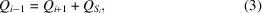
where *Q*_*i*−1_ is the inflow of the *i*th chamber, *Q*_*i*+1_ the inflow of the (*i* + 1)th chamber and 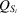 the gas flow pumped from the *i*th chamber.

Pipe conductivity is defined as the ratio of the flow through the pipe to the pressure difference between its two ends. Therefore, the equation of flow continuity balance can be reformulated as (Wang *et al.*, 2023 ▸) 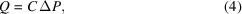

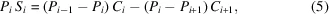
where *C*, *C*_*i*_ and *C*_*i*+1_ are the pipe conductivites, *Q* the flow through the pipe, Δ*P* the pressure difference, *S*_*i*_ the effective pumping speed of the *i*th chamber, *P*_*i*_ the pressure of the *i*th chamber, *P*_*i*−1_ the pressure of the (*i* − 1)th chamber and *P*_*i*+1_ the pressure of the (*i* + 1)th chamber.

### 2.2. Flow state analysis and conductivity calculation

The Knudsen number, a dimensionless parameter, determines the gas flow state in the pipe, involving viscous flow, molecular flow and viscous molecular flow, expressed as (Malyshev, 2020 ▸)
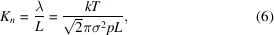

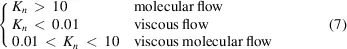
where *K*_*n*_ is the Knudsen number, λ the mean free path (m) of gas molecules, *L* the characteristic dimension (m), *k* the Boltzmann constant (J K^−1^), *T* the temperature (K), σ the effective diameter (m) of gas molecules, *p* the pressure (Pa) of the gas.

Based on the design requirements of the GAT, the working gas is N_2_ with a maximum pressure of 200 Pa, and the diameter of the AGC is 200 mm. According to the Knudsen formula (*K*_*n*_ < 0.01), the gas flow state is viscous flow in the AGC, and roughing pumps must be used (Pradervand *et al.*, 2023 ▸). Consequently, the pipe conductivity for viscous flow can be calculated by (Malyshev, 2020 ▸) 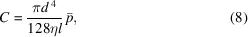
where *C* is the pipe conductivity of viscous flow (m^3^ s^−1^), η the dynamic viscosity (Pa s), 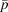 the mean pressure (Pa), *l* the length (m) of the pipe, and *d* the diameter (m) of the pipe.

Equation (8) reveals that the pipe conductivity is directly proportional to the fourth power of the differential pipe diameter and inversely proportional to the length of the differential pipe. Thus, the pipe with a smaller diameter and longer length has lower conductivity. Unfortunately, the constraints have been given by the spot size (5 mm) and the space. Considering several factors such as machining errors, installation errors and foundation settlement, the diameter of the differential pipe between the AGC and the TDPC is 10 mm, and its length is 350 mm.

According to equations (4) and (8),[Disp-formula fd8] the total flow from the AGC to the TDPC is about 787 Pa L s^−1^. This flow brings about a demand for a high-pumping-speed mechanical pump for the first stage of the DPS in the GAT. In this study, a compact TDPC was innovatively established based on 3D printing technology, as detailed in Section 3, to achieve a rapid, cost-effective, and compact differential pumping scheme for the transition from the AGC pressure (200 Pa) to the next turbomolecular pumps in the GAT.

## 3. Structure design of the TDPC

**Figure 2:**
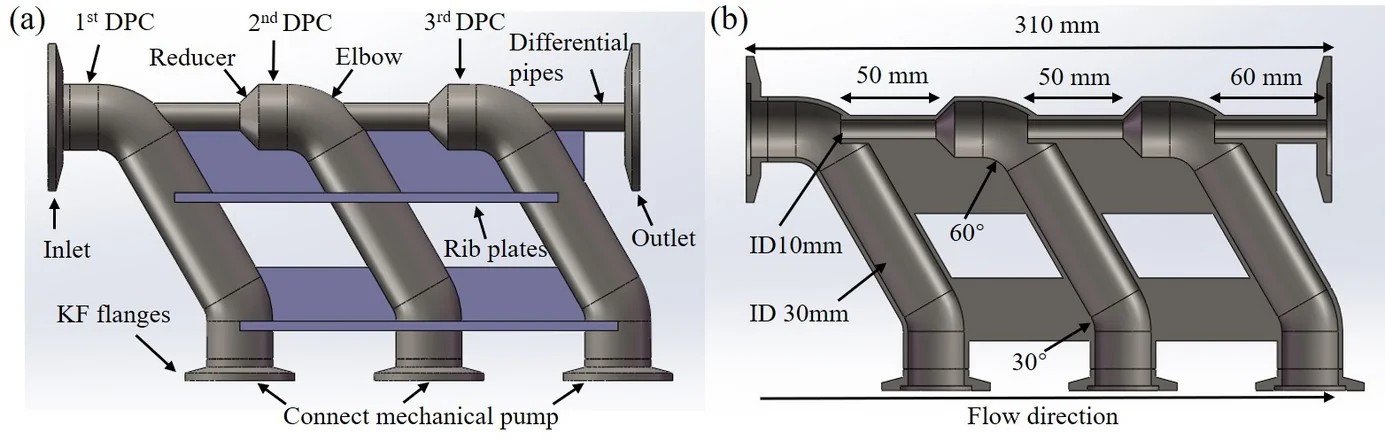
Structure of the TDPC: (*a*) front view; (*b*) section view.

The structure of the compact TDPC is displayed in Fig. 2 ▸. Similar to the differential pipe mentioned in Section 2.2, the diameter of the differential pipe in the TDPC is 10 mm, providing significant redundancy for the spot size. Following the characteristics of fluid movement along the pipe axis in viscous flow, each DPC is equipped with a special elbow to enhance pumping efficiency. The differential pipe is inserted into the elbow with a reducer between the differential pipe and the DPC to achieve a more compact structure. Notably, machining such a complex and compact structure poses significant challenges. Hence, 3D printing technology with great advantages in the fabrication of complex structures was considered (Lee *et al.*, 2023 ▸).

In a rough vacuum region, KF flanges are widely employed to connect vacuum components. Nevertheless, 3D printing technology may induce unevenness of the surface on KF flanges, which could compromise the vacuum seal. Consequently, machining allowances for KF flanges are reserved during fabrication to benefit subsequent machining, while some ribs are incorporated into the DPC to provide structural reinforcement and facilitate positioning and clamping during the machining procedure.

## 4. Numerical simulation

The differential performance of the TDPC should be evaluated before processing. Attributed to the variability of the pumping speed with the inlet pressure, it is difficult to acquire the pressure distribution by theoretical calculations. The Knudsen number in Section 2.2 suggests that the gas flow state is viscous flow, which adheres to the continuous medium hypothesis. Hence, the pressure distribution of the TDPC can be calculated by numerical simulation based on the Navier–Stokes equation expressing conservation of momentum and mass (Yang *et al.*, 2017 ▸).

**Figure 3:**
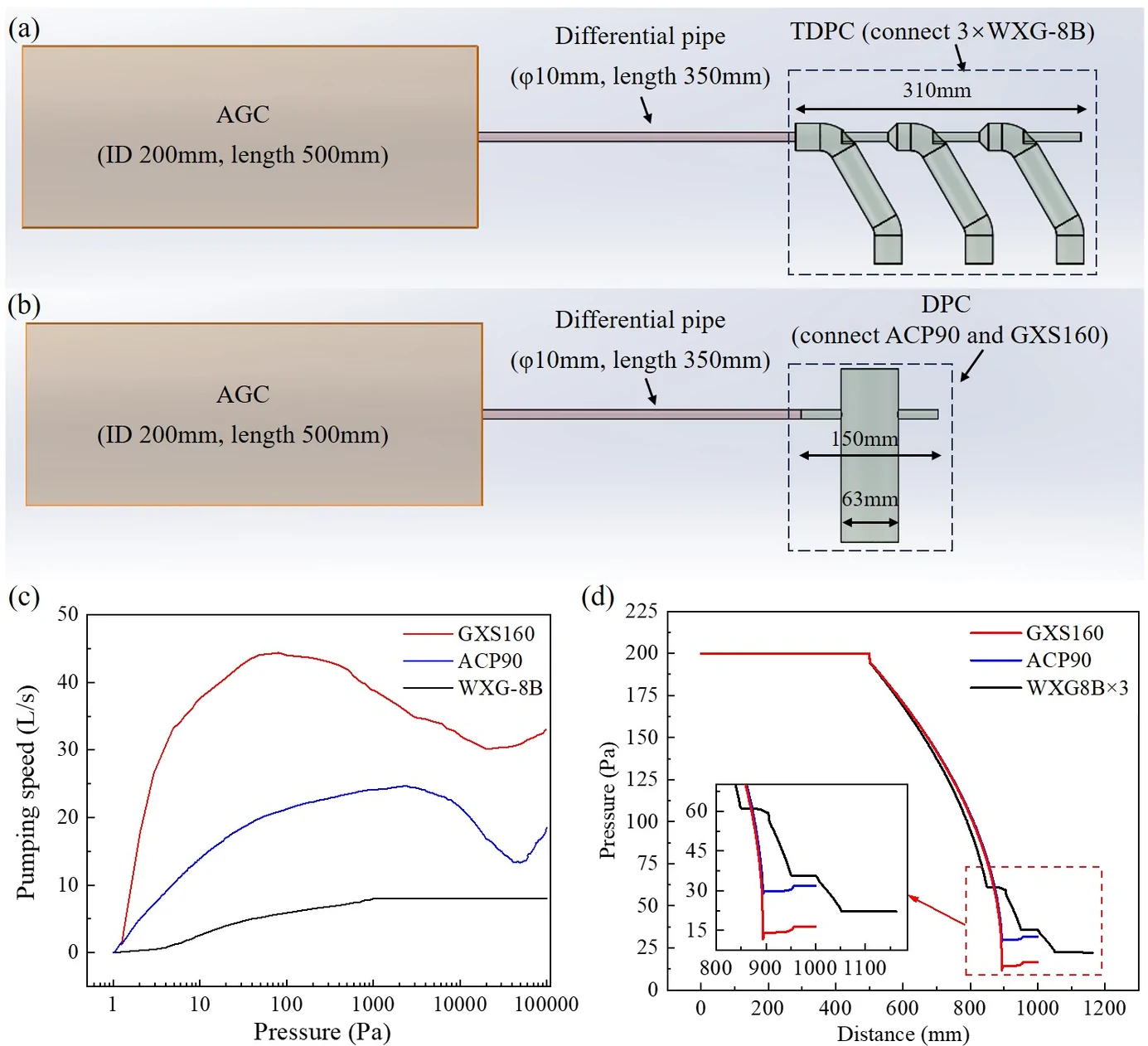
(*a*) Inner flow domain of the TDPC; (*b*) inner flow domain of the DPC; (*c*) pumping speed curves; (*d*) pressure distribution.

Half of the AGC model and one-sided DPS were considered for simulation owing to the symmetry of the GAT. Figs. 3 ▸(*a*) and 3 ▸(*b*) illustrate the two inner flow domains utilized for numerical simulation to compare the differential performance of the TDPC with three scroll pumps (WXG-8B, SKY Technology Development Co., Ltd) and the conventional DPC with high-pumping-speed mechanical pumps (ACP90, scroll pump, Pfeiffer; GXS160, screw pump, Edwards). The effects of photon–gas interactions were disregarded, and the pressure of the AGC along with the pumping speed served as the boundary conditions for the FEA, as depicted in Fig. 3 ▸(*c*). Based on these assumptions and the inner flow domains, the pressure distributions along the centerline were calculated, as illustrated in Fig. 3 ▸(*d*).

The simulation results reveal that the largest pressure drop occurs in the conventional DPC with the GXS160 (16.4 Pa), followed by the TDPC with three WXG-8Bs (22.3 Pa), and finally the conventional DPC with the ACP90 (31.8 Pa). Notably, the maximum pumping speed of the ACP90 is three times greater than that of the WXG-8B. Nonetheless, the differential performance of the conventional DPC with the ACP90 is inferior to that of the TDPC with three WXG-8Bs. While the maximum pumping speed of the GXS160 is 5.5 times that of the WXG-8B, the differential performance between the two is not significantly different. The main reason for this phenomenon is that a multistage pump scheme can provide a larger inlet pressure for each stage to achieve better pumping efficiency and differential performance, thereby avoiding the problem of a significant drop in effective pumping speed for a single pump under low pressure.

While the GXS160 offers a higher peak pumping speed, its overall differential performance (16.4 Pa versus 22.3 Pa) is only marginally better. Employing three smaller, commercially available WXG-8B pumps yields a comparable differential performance with about 50% less energy consumption, 57% less weight and 20% lower capital cost, as detailed in Table 1 ▸. Notably, the TDPC exhibits a longer axial dimension (310 mm) compared with the conventional DPC (150 mm). Nevertheless, the DPC + GXS160 system requires a heavy, bulky pump (305 kg) with a dedicated supporting frame and floor anchors. In contrast, the 3D-printed TDPC can be connected to three mechanical pumps through bellows, and the mechanical pumps could be placed directly on the ground without additional reinforcement. Consequently, the entire vacuum system becomes more spatially flexible and is particularly suitable for space-constrained or low-energy laboratory environments.

**Figure 4:**
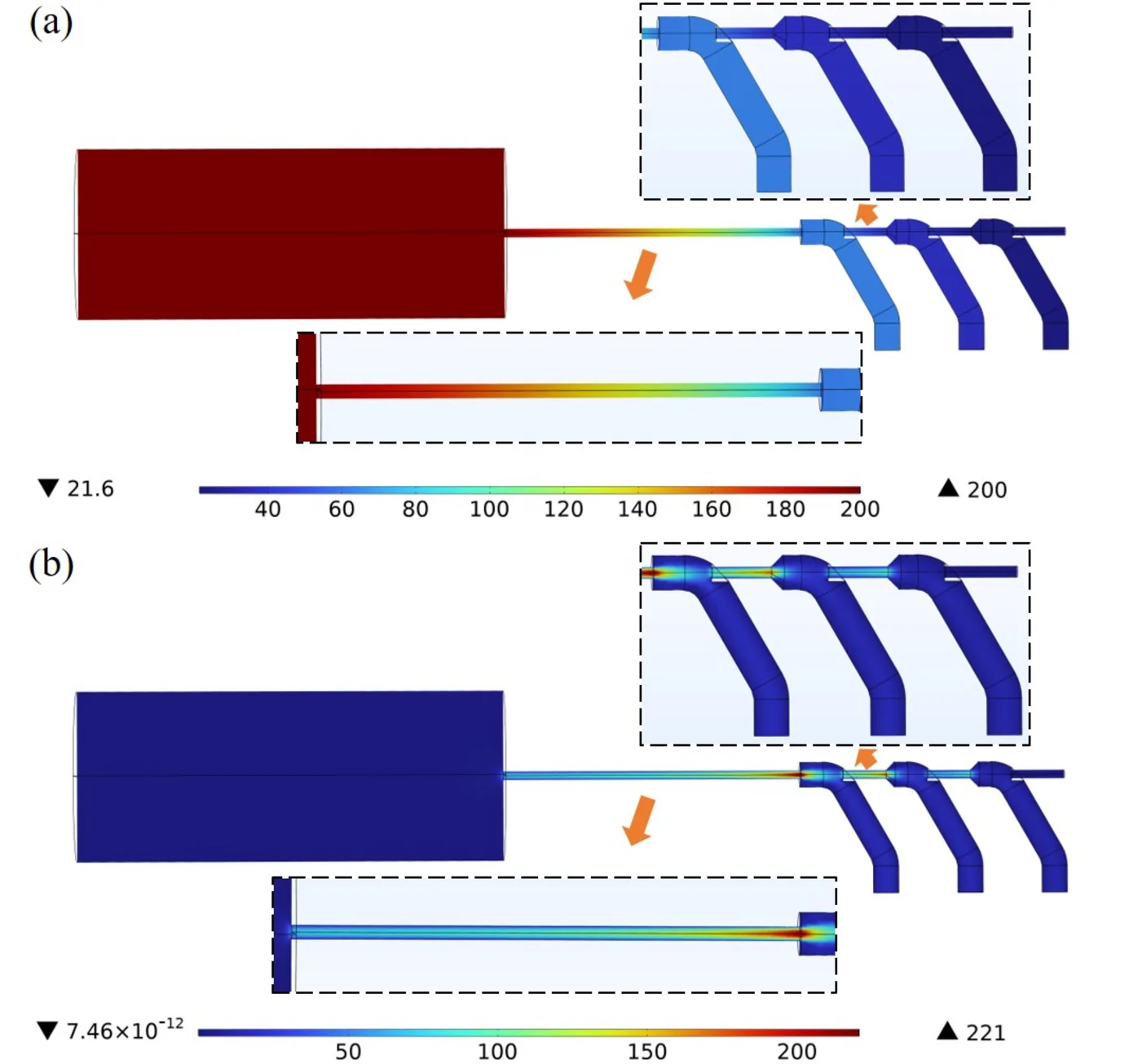
Simulation results with an AGC pressure of 200 Pa: (*a*) pressure distribution; (*b*) velocity distribution.

Fig. 4 ▸ displays the pressure and velocity distributions within the cross-section of the inner flow domain with the TDPC. The velocity distribution clarifies that the flow remains subsonic, consistent with the results of Wang *et al.* (2024 ▸). The increase in velocity at the different pipes is primarily affected by the decrease in area based on the flow conservation and is consistent with the results of Pradervand *et al.* (2023 ▸). Additionally, the pressure drop starts from the differential pipe, with the pressure dropping to 60 Pa at the first DPC, 35.8 Pa at the second DPC and 22.3 Pa at the third DPC, as observed from the pressure distribution. The overall pressure drop ratio is around 0.1. Generally, a lower pressure at the third DPC is more conducive to the stable operation of the next differential pump (Hipace 450, turbomolecular pump, Pfeiffer). At an operating pressure of 0.1 Pa, its pumping speed reaches a maximum, and the pump operates stably. In the future, the bellows (length 100 mm) should be placed between the TDPC and the next stage of the turbomolecular pump vacuum chamber (length of flange connection pipe, 40 mm). Hence, the total length of the differential pipe is 200 mm. In Fig. 2 ▸, the length of the differential pipe downstream of the third DPC is 60 mm. As calculated from equations (6) and (7), the Knudsen number is about 0.023, and the gas flow state is viscous molecular flow. Additionally, the pipe conductivity is about 1.5 L s^−1^, reflecting that the gas flow is 33.45 Pa L s^−1^ (22.3 Pa × 1.5 L s^−1^). Based on the pumping speed of the Hipace 450 (380 L s^−1^ for N_2_), the pressure on the next turbomolecular pump vacuum chamber is calculated to be about 0.088 Pa, which enables stable operation for the turbomolecular pump. Notably, the simulations in this study ignore the pumping speed of the differential pipe downstream of the third DPC. The pressure at the third DPC is even lower when the next turbomolecular pump is connected. Furthermore, the pressure on the next turbomolecular pump vacuum chamber is less than 0.088 Pa, which is better for the operation of the turbomolecular pump.

**Figure 5:**
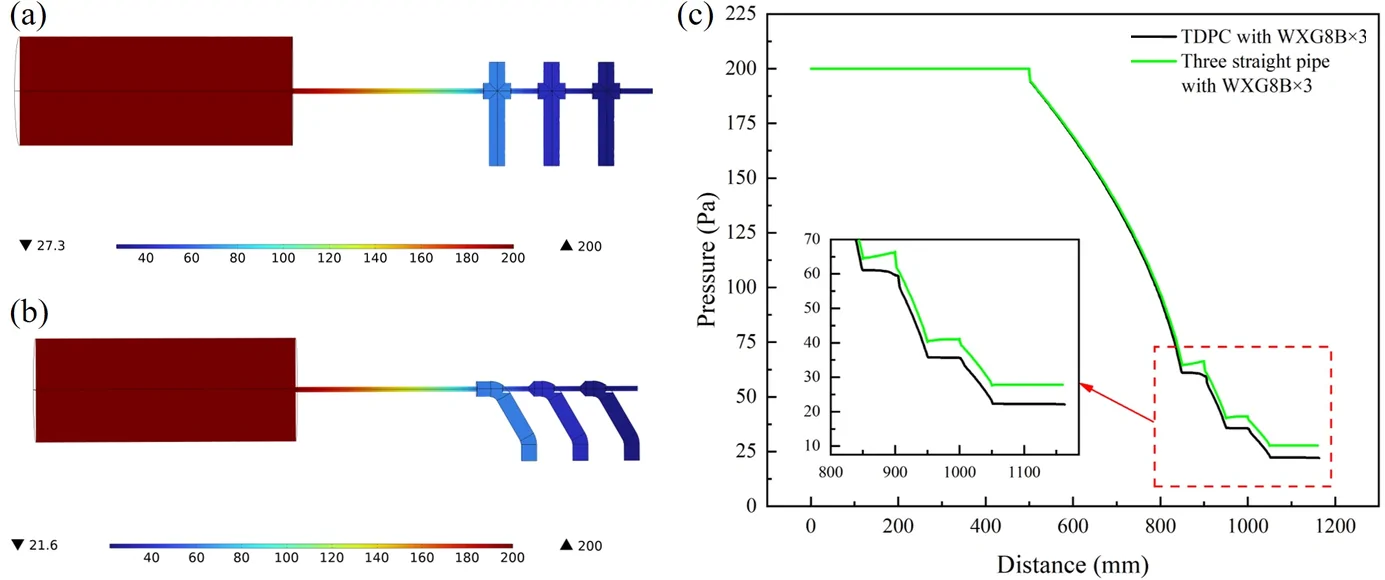
Pressure distribution: (*a*) straight pipe; (*b*) TDPC; (*c*) comparison.

For viscous gas flow, the flow lines change with the varying shape of the pipe. The design of the special elbow promotes gas pumping away and improves pumping efficiency. The differential performance of the straight pipe and the TDPC is compared, as illustrated in Fig. 5 ▸. The straight pipe and the TDPC have the same length of 310 mm. Meanwhile, the height between the flanges connected to the pumps and the centre of the differential pipe is consistent. Figs. 5 ▸(*a*) and 5 ▸(*b*) display cross sectional views of pressure distributions for the straight pipe and the TDPC, respectively. Fig. 5 ▸(*a*) exhibits the pressure distributions along the beamline. The pressure at the end of the straight pipe (27.8 Pa) is higher than that at the TDPC (22.3 Pa), revealing that the TDPC has better differential performance compared with the straight pipe, attributed to the special elbow.

## 5. Manufacture of the TDPC and experimental setup

### 5.1. Manufacture of the TDPC

**Figure 6:**
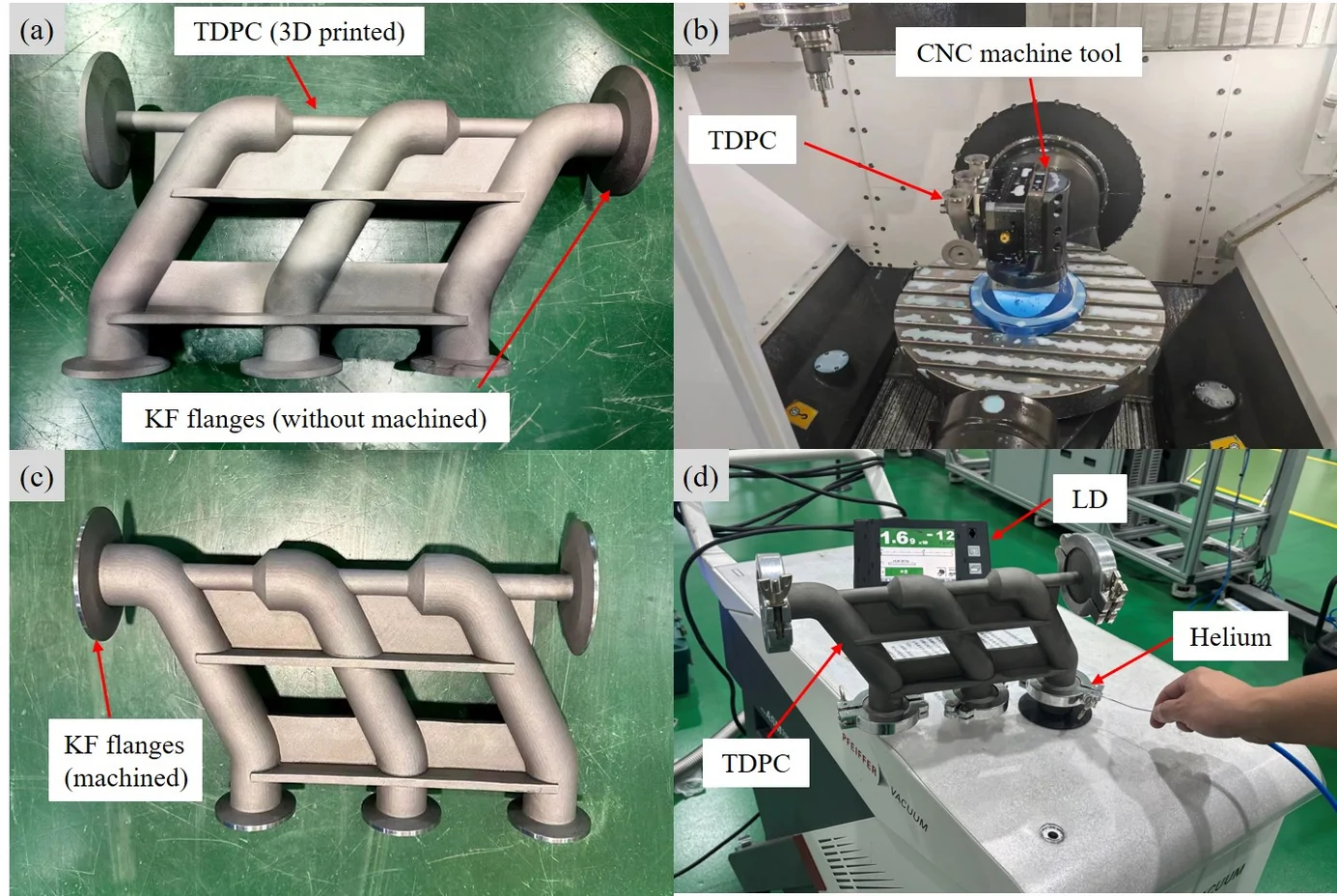
Manufacture of the TDPC: (*a*) printed by a 3D printer; (*b*) machined by a CNC machine tool; (*c*) vacuum-cleaned and dried; (*d*) vacuum leak detection.

The TDPC was manufactured from 316L stainless steel by 3D printing, requiring approximately 90 h to complete [Fig. 6 ▸(*a*)]. Subsequently, the printed raw part was stress-free annealed, and the KF flanges were machined by CNC to achieve an acceptable vacuum seal [Fig. 6 ▸(*b*)]. In this study, the straightness and inner diameter are the key parameters for the three differential pipes. Fabrication accuracy was ensured by fabricating two custom-made solid cylindrical gauges: a pass-through gauge (diameter 9.8 mm) and a stop gauge (diameter 10.2 mm). Both gauges were machined with a straightness of 0.1 mm, guaranteeing that they served as reliable references for evaluating both diameter and straightness. The pass-through gauge was inserted into and passed through the TDPC without any noticeable resistance. This result confirms that the inner diameter of each pipe was greater than 9.8 mm and that the pipe straightness was strong enough to allow a straight gauge to traverse the full length freely. Additionally, the stop gauge was inserted from both ends separately while being blocked at the entrance and could not proceed further. In other words, the inner diameter was less than 10.2 mm. Taken together, the TDPC achieved sufficient accuracy in inner diameter and straightness. Furthermore, considering the spot size (5 mm), significant redundancy was retained for the diameter of the differential pipe (10 mm) in the TDPC. Therefore, it met the requirements.

Afterward, the TDPC was vacuum-cleaned and dried before measurement [Fig. 6 ▸(*c*)]. A helium mass spectrometry leak detector (LD, Adixen ASM390, Pfeiffer) was utilized to measure the vacuum leak rate of the TDPC, yielding a vacuum leakage rate of 1.69 × 10^−12^ Pa m^3^ s^−1^ [Fig. 6 ▸(*d*)]. This value is within the acceptable range for high-vacuum systems employing elastomer-sealed KF flange connections (Poynton *et al.*, 2025 ▸), confirming that the 3D-printed metal body is free from detrimental porosity.

### 5.2. Experimental setup

**Figure 7:**
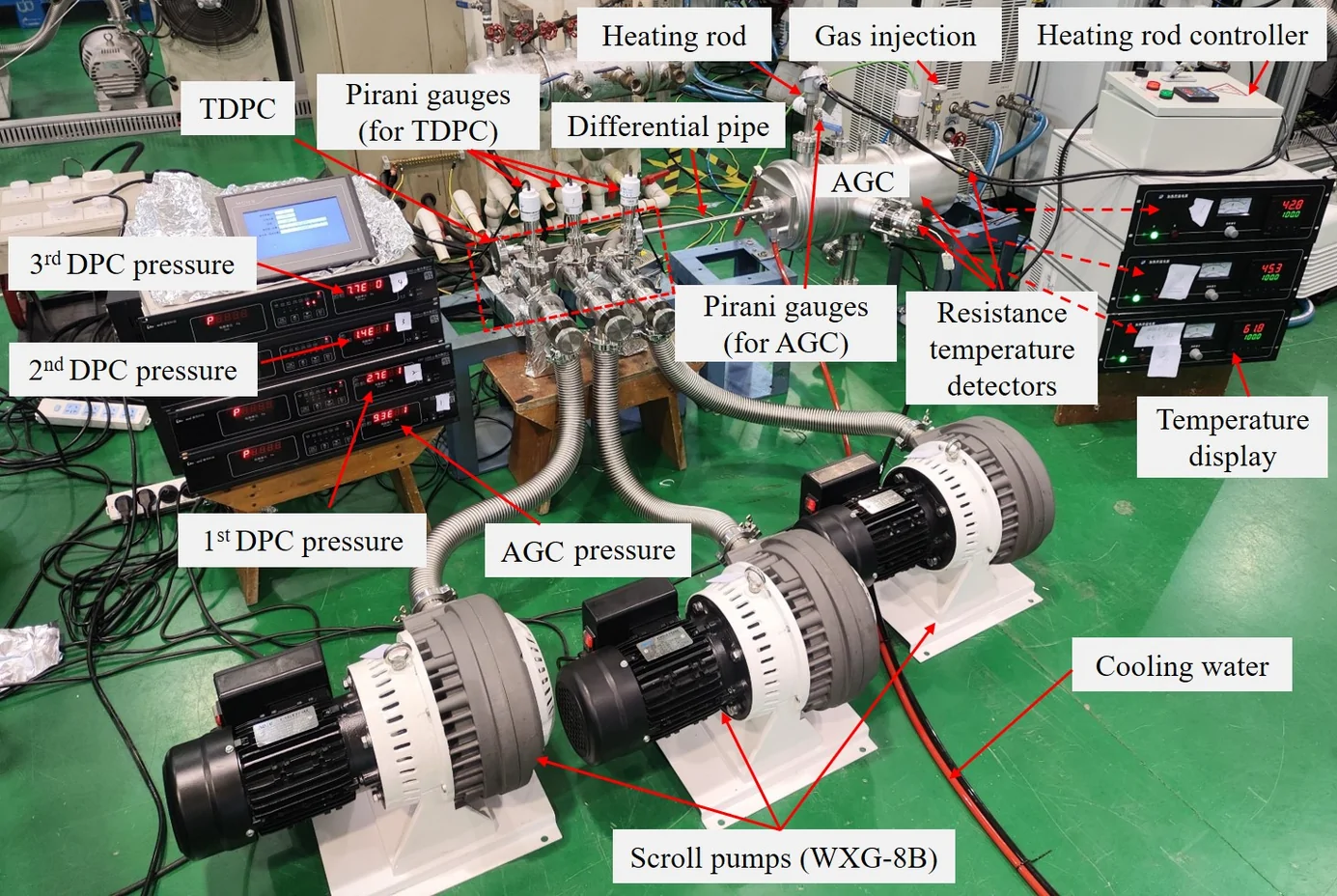
The experimental system for testing the differential performance of the TDPC.

An experimental system, consisting of the AGC, differential pipe, and the TDPC, was designed to evaluate the differential performance of the TDPC (Fig. 7 ▸). N_2_ was injected into the AGC, and the AGC pressure was precisely adjusted through the flow controller or microleakage valve. Additionally, the TDPC was connected to the three scroll pumps (WXG-8B, SKY Technology Development Co., Ltd) for pumping, with pressure measurements taken by Pirani gauges (ZJ52, ChengDu Reborn Electronics Co., Ltd).

The AGC features four gas injection positions to assess the differential performance of the TDPC. The interaction of photon beams with gas leads to an increase in temperature (Yang *et al.*, 2017 ▸). Therefore, a heating rod (up to 600°C) was installed in the AGC near the differential pipe to evaluate the differential performance of the TDPC under different heating temperatures. Meanwhile, three resistance temperature detectors (RTDs) were placed within the AGC to monitor the temperature distribution. For the purpose of preventing overheating of the AGC, water cooling channels were incorporated into the outer walls of the AGC near the heating rod.

## 6. Results and discussion

### 6.1. Comparison between experiment and numerical simulation

**Figure 8:**
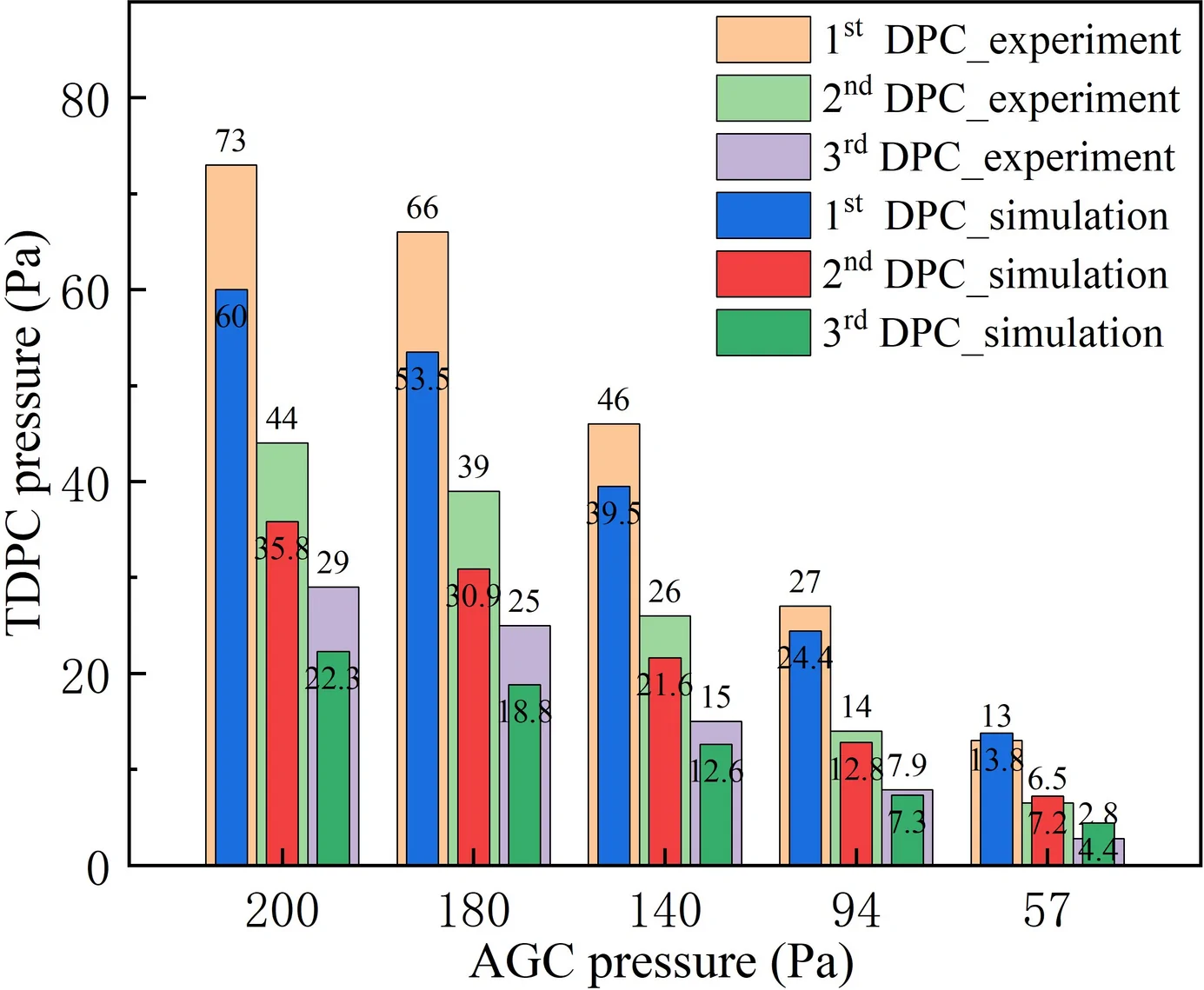
Comparison of the pressure distribution of the TDPC between experimental and simulation results.

The differential performance of the TDPC with different AGC pressures was calculated based on the method mentioned in Section 4. The experimental results were compared with the corresponding simulated results, as illustrated in Fig. 8 ▸.

The experimental and simulation results are more consistent as the AGC pressure decreases, and the experimental pressures of each DPC of the TDPC are slightly larger than those of the simulation, except for the AGC pressure of 57 Pa. Additionally, the absolute error (*P*_experiment_ − *P*_simulation_) of each DPC in the TDPC gradually decreases in the flow direction, with a maximum value of 13 Pa. The relative error [(*P*_experiment_ − *P*_simulation_)/*P*_experiment_] of each DPC in the TDPC also gradually decreases with the decreasing AGC pressure, remaining within 25%. Both the experimental and simulation results have demonstrated effective differential performance of the TDPC and good consistency. Considering the reduction of effective pumping speed due to the bellows and the measurement error of vacuum gauges, the difference between experimental measurement and simulation is acceptable for engineering applications, verifying the practicability of the simulation.

### 6.2. Effect of different gas injection positions without heating

**Figure 9:**
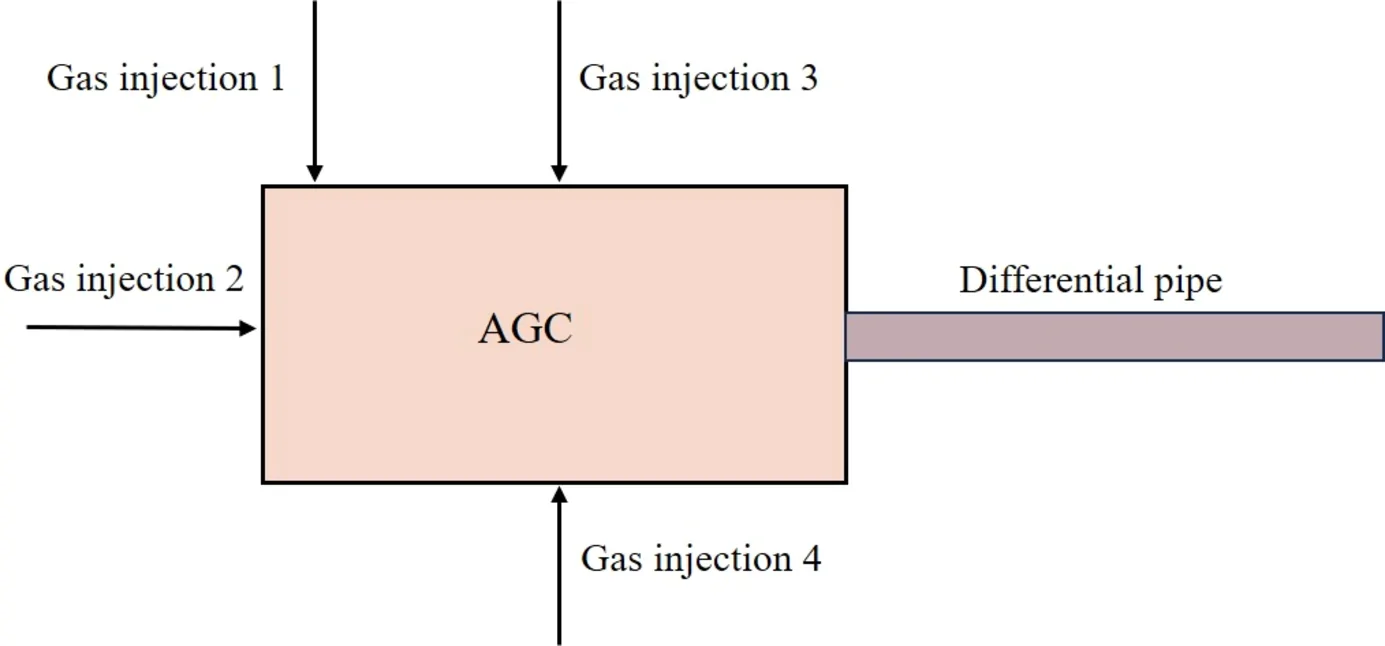
Four gas injection positions on the AGC.

In future installations, the gas injection position may deviate from the centre of the AGC. Under this consideration, the differential performance of the TDPC was measured at different gas injection positions (Fig. 9 ▸) without heating based on the experimental system displayed in Table 2 ▸. The achievable maximum AGC pressure is approximately 100 Pa, attributed to the limit of the flow controller (0–100 sccm). Therefore, the variable leak valve was used to achieve the AGC pressure of 200 Pa.

Each DPC pressure in the TDPC at different gas injection positions is the same under the same AGC pressure. In other words, the gas injection position exerts little effect on the differential performance of the TDPC. Thus, the consumed gas flow is also consistent because of the consistent DPC pressure of the TDPC and the same pumps. Moreover, each DPC pressure in the TDPC exhibits a nearly linear increase as the AGC pressure increases linearly. When the AGC pressure reaches 200 Pa, the third DPC pressure is about 30 Pa, which facilitates operation of the next differential pump (turbomolecular pump). This result validates the feasibility of the TDPC in the DPS for the GAT.

### 6.3. Effect of different heating temperatures

**Figure 10:**
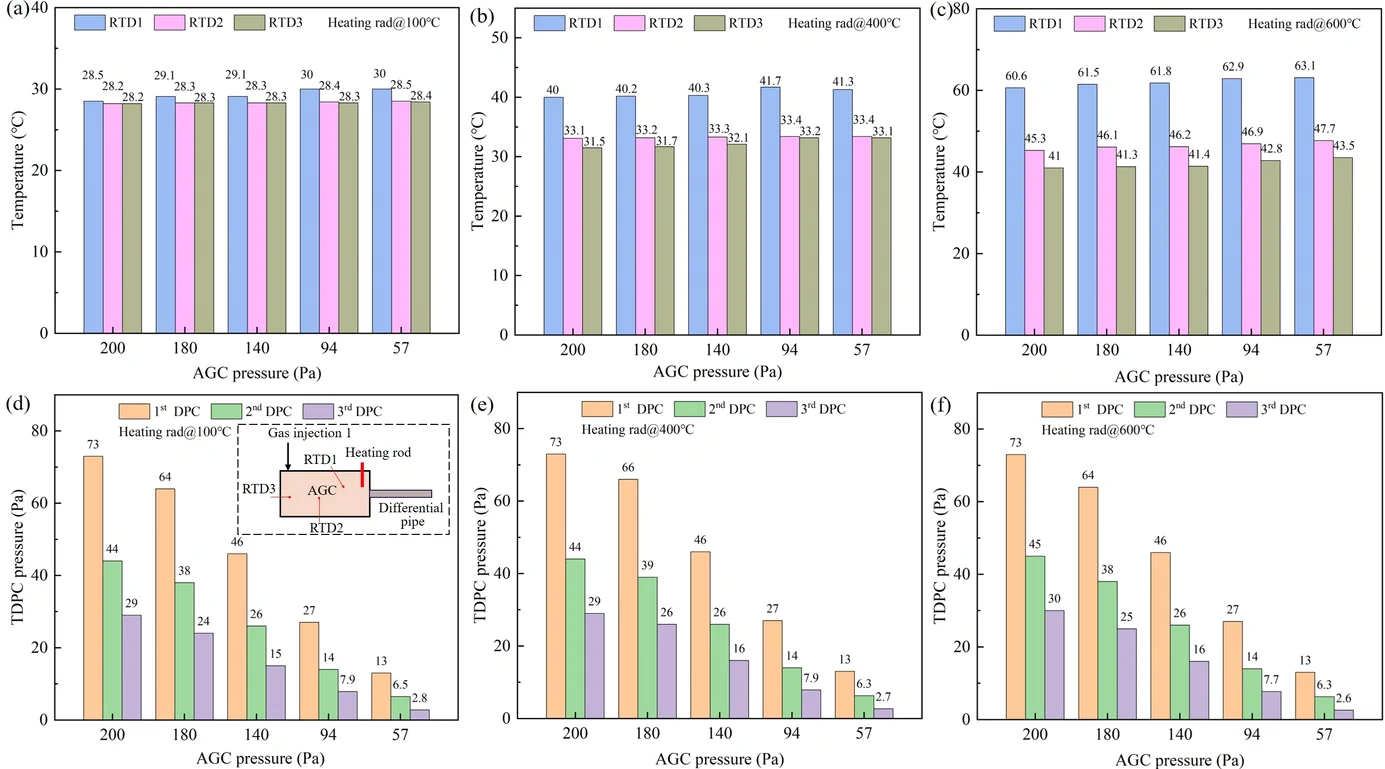
Temperature of the RTDs on the AGC and the pressure distribution of the TDPC and with different heating temperatures: temperature of the RTDs at heating temperature of (*a*) 100°C, (*b*) 400°C and (*c*) 600°C; pressure distribution of the TDPC at heating temperature of (*d*) 100°C, (*e*) 400°C and (*f*) 600°C.

Given the negligible effects the gas injection position on the differential performance of the TDPC according to Section 6.2, only gas injection 1 was measured with heating. The heating rod was adopted to simulate the temperature rise near the differential pipe arising from the interaction of gas and photons. The cooling water was supplied to prevent excessive temperature and leakage of flanges. Fig. 10 ▸ depicts the temperature of the RTDs on the AGC and the pressure distribution of the TDPC with different heating temperatures.

Three RTDs were installed on the AGC to measure its inner temperature distribution, as illustrated in Figs. 7 ▸ and 10 ▸(*d*). As observed from the figures, the temperatures of all RTDs slightly increase as the AGC pressure decreases at the same heating temperature. This phenomenon primarily stems from the reduction in heat transfer from the outer cooling water because of the decreased AGC pressure, which promotes heat transfer within the AGC. With the increasing heating temperature, the temperature change observed at RTD1 is the most significant under the same AGC pressure, followed by RTD2, and then RTD3. This is remarkably influenced by the distance from the heating rod. Overall, RTD3 remains at a relatively low temperature, despite the high heating temperatures. In other words, the temperature drops significantly over a short distance, and the heat is essentially concentrated near the heating rod, in line with the calculations of Wang *et al.* (2024 ▸).

Figs. 10 ▸(*d*), 10 ▸(*e*), and 10 ▸(*f*) illustrate the pressure of the AGC and each DPC in the TDPC with different heating temperatures. The results suggest that the variation in pressure of each DPC in the TDPC remains minimal as the heating temperature increases. Thus, the heating temperature exerts little effect on the differential performance of the TDPC. Since the pumping speed of scroll pumps mainly decreases with increasing working temperature (Zhang *et al.*, 2023 ▸), the heat generated from the AGC is minimally transferred to the scroll pumps. Additionally, the pressure drops occur at the differential pipe [Fig. 4 ▸(*a*)], which generates the Joule–Thomson effect, leading to an increase in velocity and a significant decrease in temperature (Kumar *et al.*, 2025 ▸). Furthermore, the Joule–Thomson effect further hinders the heat transfer from the AGC to the scroll pumps, validating the little effect of heating temperature near the differential pipe on differential performance of the TDPC. The results regarding heating temperature on the AGC are crucial for the future design of the DPS in the GATs.

## 7. Conclusion

In this study, a compact TDPC was innovatively designed and fabricated by 3D printing technology, serving as an interface between the AGC and the next turbomolecular pumps of the GAT. The FEA methods were employed to evaluate the pressure distribution of the TDPC with three low-speed scroll pumps (8 L s^−1^) and demonstrate its favourable differential performance (from 200 Pa to 22.3 Pa). The vacuum leakage rate was 1.69 × 10^−12^ Pa m^3^ s^−1^ for the TDPC, and the simulation results show good consistency with the experimental results (presenting a relative error within 25%), which is acceptable for engineering applications considering the reduction of effective pumping speed due to the bellows and the measurement error. Additionally, the experimental results revealed that the pressure was reduced from 200 Pa to 29 Pa. This facilitated the operation of the next differential pump (turbomolecular pump) while validating the feasibility of the TDPC in the DPS. Furthermore, the gas injection position of the AGC and the heating temperature (600°C) near the differential pipe exert little influence on the differential performance of the TDPC. This study introduces a novel DPC concept, verifies its potential for application in DPS, and offers a useful reference for the design of DPS systems in GATs.

## Figures and Tables

**Table 1:** Performance parameters of the mechanical pumps (WXG-8B, GXS160)

	WXG-8B	GXS160
Maximum pumping speed (L s^−1^)	8	44.5
Weight (kg)	44	305
Dimension (l × w × h) (m)	0.485 × 0.333 × 0.357	1.092 × 0.39 × 0.568
Maximum power consumption (W)	850	5000
Maintenance period (year)	2	2
dB (A)	65	65
Price ($)	∼4000	∼15000
Differential performance (simulation) (Pa)	22.3	16.4

**Table 2:** Pressure distribution of the TDPC at different gas injection positions without heating

Gas injection	Pressure AGC (Pa)	Pressure 1st DPC (Pa)	Pressure 2nd DPC (Pa)	Pressure 3rd DPC (Pa)
1	200	73	44	29
	180	66	39	25
	140	46	26	15
	94	27	14	7.9
	57	13	6.5	2.8

2	200	74	46	30
	180	64	39	25
	140	46	27	16
	94	27	14	7.9
	57	14	7	3.2

3	200	73	45	30
	180	64	38	25
	140	48	28	17
	94	27	15	8.3
	57	14	7	3.3

4	200	74	45	30
	180	62	37	23
	140	48	28	17
	94	27	14	7.9
	57	13	6.5	3
